# Pragmemes revisited. A theoretical framework

**DOI:** 10.3389/fpsyg.2024.1329291

**Published:** 2024-01-31

**Authors:** Alessandro Capone, Roberto Graci

**Affiliations:** Department of Cognitive Sciences, Psychology, Education and Cultural Studies (COSPECS), University of Messina, Messina, Italy

**Keywords:** pragmemes, pragmatics acts, societal pragmatics, speech act, language game

## Abstract

In this paper, we take up an old issue that of pragmemes, broached by Mey and further explored by Capone. It is not easy to define pragmemes and distinguish them sufficiently from speech acts (units of language use broached by Austin and Searle) or from Wittgensteinian language games or from macro speech acts (see van Dijk on macrostructures) or from Goffman’s scripts. The best idea we could develop about pragmemes is that they instantiate the triple articulation of language, proposed by Jock Wong; being essentially composed of phonological-syntactic units, that have a certain content relative to a social situation and to a certain culture, pragmemes express a certain function (or illocutionary force), like, e.g., modifying society or some aspect of it. They are part of a chapter that can be called either “societal pragmatics” or “emancipatory pragmatics,” to use the words by Mey. In fact, knowledge of how language is used to diminish the rights of people and to propagate the “status quo” may be instrumental to give rights and power to ordinary human beings who are oppressed by political and economical structures.

## 1 Map of the paper

This article provides a comprehensive overview of recent advancements in the theory of pragmemes. According to definition of Capone (2005), a pragmeme is a situated speech act that aims to modify situations and participants’ roles. This act can yield diverse outcomes, such as information exchange, social gratifications, and the formulation of rights and duties. The article focuses on three key aspects: (1) the impact of language usage conventions on pragmemes, (2) the influence of cultural values on linguistic forms within pragmemes, and (3) the intricate interplay of social and environmental conditions in interpreting speech acts. Section 4 deals with the first point. The role of usage conventions in determining pragmemes is evident in the so-called Situational-Bound Utterances (SBUs), i.e., prefabricated pragmatic units reiterated in standardized communicative situations (greetings, introductions, requests, etc.). Section 5 takes a focused approach, tracing the evolution of a specific SBU—the question “Vieni?” (“Will you come?”) uttered by Italian teachers during classroom assessments. Sections 6–8 shed light on the profound impact of cultural values in shaping particular linguistic forms. In particular, section 6 examines the resonating power of Obama’s iconic phrase, “Yes, we can.” Not only does this utterance expresses the enthusiasm of the ex-president of the United States but also encapsulates American ideals such as optimism, hard work, and equal rights. Instead, sections 7 and 8 analyze specific Italian linguistic forms within standardized contexts. Their understanding also demands an in-depth exploration of Italian cultural norms and values. Section 9 delves into the intricate interplay of social and environmental conditions in interpreting speech acts. It dissects a controversial utterance by former President Trump to FBI Director Comey (“I hope you will let Flynn go”). Unlike past scenarios, the meaning of this utterance is not determined primarily by the force of usage conventions or cultural values. Here, pragmatic inferences, predominantly driven by situational context and social roles, guide the interpretative processes. Section 10 meticulously investigates another scenario where situational conditions take priority over other factors in interpreting a situated speech act: names uttered in isolation. Section 11 revisits the symbiotic relationship between form, meaning, and culture, advocating for an encompassing approach in linguistic analysis. It is good to consider the cultural context within which certain linguistic forms are used to obtain a more realistic picture of language. Section 12 directs attention toward two fundamental aspects of the theory of pramemes. The first aspect, as illuminated by Oishi (2016), pertains to general conditions that underpin the agreement on the discursive status of a situated speech act. The second aspect delves into the significance of individual dispositions, emphasizing their pivotal role in the production and interpretation processes within a pragmeme. This article aims to give readers comprehensive and contemporary insight into the multifaceted theory of pragmemes.

## 2 The importance of pragmemes

In exploring the literature on pragmemes, we pursue two main aims. First, we want to help readers understand the importance of this concept for the revitalization of a field of linguistics that has long been overshadowed: socio-anthropological linguistics. Second, we seek to elucidate the role of the pragmeme within a language theory dedicated to understanding social action.

The first aspect revolves around the challenge of emancipating modern linguistics from the constraints of formal paradigms. Since the 1960s, the Chomskyan approach has undeniably dominated language studies. Its proponents have set themselves the ambitious goal of unraveling the regularities of natural languages through abstract and mathematical representations of the generative systems of rules underpinning their functioning. Specifically, formal scholars posit that syntactic rules are somewhat independent and analyzable in isolation from other sources of information (e.g., lexical or contextual data)—an idea referred to as “the autonomy of syntax” (Croft, 1995). The formal paradigm appears in direct contrast to holistic language approaches (Capone, 2005). It sanctions the analysis of syntax as a self-contained system governed by its own laws, divorced from other components of human language. In its most radical manifestation, the formal approach posits that highly general syntactic processes, common to all languages, are innate—they are activated independently of the environment and interact with parametric choices. This perspective views human language not as a conventionally shaped phenomenon but as one constrained by biological determinants (up to a point this point of view can be accepted, provided that we do not claim that this is all that linguistics is about). We will not delve into the complexities of this idea here (for a more extensive discussion, see Capone, 2010a,b,c,d). Instead, we want to underscore that the exclusive emphasis of formal linguistics on biology and syntax as an independent system of rules had profound consequences on the direction of linguistic research at the end of the last century. Scholars, except for linguists with anthropological training, for an extended period, displayed a tangible tendency to neglect the social dimension of language acquisition. Chomskyan linguistics has firmly drawn a distinction between the role of language in articulating thoughts and its communicative functions (Jakobson, 1995). While the former took center stage in linguistic investigations, communicative functions were often treated as optional and unworthy of in-depth study. We do not intend to dismiss the ongoing relevance of insights from Chomskyan linguistics (since, without certain biological structures passed down from one generation to the next, certain cognitive abilities would not be feasible). However, it is equally important to explore how humans have acquired the non-universal, non-biologically innate aspects of language, which also appear to be integral to sociocultural inheritance. Those who go beyond the examination of the “internal” organization of language recognize its extensive communicative power and its indispensable role in human life (see, for example, Pinker and Jackendoff, 2005). Once we neglect the social dimension of language, including its various and multifaceted functions, the interplay between the expression of thought and the social and institutional aspects of society, as well as the propagation and transmission of culture through language, we are left with a notably impoverished and sterile vision of linguistics (Capone, 2010b, 2023).

Given that the theory of pragmemes signifies a revival of anthropological approaches to language that go beyond formal ones, it is essential to elucidate the role of the pragmeme as a unit of analysis within the spectrum of theories of language use. Completing this task proves challenging, given the proliferation in the philosophical domain of analogous units of analysis, such as “speech acts,” “language games,” or “utterances.” Indeed, one might question whether merely situating a speech act in a context is sufficient to introduce a novel concept into the realm of pragmatics. Could it be that a pragmeme is synonymous with Wittgenstein’s language game? The crux lies in the fact that while the concept of the pragmeme shares certain similarities with both speech acts and language games, it performs a function that these constructs cannot fully encompass. To begin with, a speech act is a unit of analysis that tends to be overly “philosophical.” It corresponds to a sentence with some context, frequently artificially constructed (see Mey, 2001; Capone, 2020). In its initial formulation, a speech act is a sentence spoken with a certain force, exerting an influence on society by altering its deontic attributes. For instance, a request entails an obligation for the hearer to perform a certain action (barring any impediments hindering the fulfillment of this obligation). On the other hand, a promise grants the hearer the right to anticipate the speaker’s commitment to a particular action (provided it lies within the speaker’s capability). While these categories are useful, grasping the mechanics of speech acts becomes unattainable without situating them in textual sequences and without accounting for the critical factor of context. When we “embed” an utterance in a dialogic sequence within an actual scenario, we gain access to a multitude of macroscopic and microscopic contextual signals that aid in its interpretation. Dascal (2003) sees the contribution of context to interpretation as an interplay of clues and cues, as the author explicates:

Our analysis thus suggests that in interpreting a text the reader draws on two different kinds of information: (a) clues, both co-textual and contextual, which will lead him toward the determination of utterance meaning and speaker’s meaning; (b) cues, which help him to distinguish between opacity and indirectness. The cue for opacity is the need for gap-filling, whereas the cue for indirectness is a mismatch between utterance meaning and second channel information (Dascal, 2003, 183).

Texts frequently employ expressions or intonational devices to indicate how a segment of speech should be interpreted and to articulate the speaker’s intention in connection with it. Dialogues often utilize signals to isolate a functional unit comprising multiple utterances (such as a story, a joke, or an argument) and prescribe the hearer’s behaviors in relation to this unit (for instance, refraining from interrupting a lecture, joke, or story). However, these elements find limited consideration in the theory of speech acts.

Second, diverging from the speech act (as normally intended by philosophers), a pragmeme is a composite entity shaped by both conventional and cultural aspects, if we adhere to tripartite framework of form-meaning-culture of Wong (2010). Cultural elements are integral and not incidental to linguistic analysis. In the upcoming pages, we will provide several examples of pragmemes, demonstrating that a thorough understanding necessitates a deep knowledge of languages in culture. We highlight that the value of a given expression is not solely derived from its compositional meaning but also from its usage in specific ways and contexts by speakers who share a common sociocultural background. While speech acts can be translated between languages, the cultural underpinnings of pragmemes make them a challenge to translators’ efforts.

When we explore the study of social actions, the analysis should not center on individual utterances or statements, but rather on goal-oriented sequences of utterances. In this context, the concept of pragmeme is inherently dialogic. To fully comprehend any utterance, it is imperative to situate it within an interactive exchange. Simultaneously, each interactive exchange is guided by rules acquired and passed down through anthropological practices. In this regard, the notion of language game closely aligns with the holistic unity we have in mind. Typically accompanied by rules of use, the language game is essential as it clarifies what actions are permissible or not within the domain of language, encompassing various social constraints. These usage rules play a pivotal role in both production and interpretation, often expressed as “utterance X should be considered as Y in context Z.” Nevertheless, there is a crucial element missing from the concept of language game, and it pertains to the pragmeme’s generative capacity to establish new norms within a given scenario [for example, a student who asks for permission to eat during a class has created a precedent and has established a new norm if that behavior is accepted in that class and then in other classes (see Capone, 2010c, 2010d)]. The rules within a language game guide us on how the game should be played, emphasizing similarities. While we may encounter manifold situations, the clarity of these rules ensures that we understand the language game, specifying the appropriate actions for each circumstance. Conversely, pragmemes, such as lecturing, selling at the market, reciting a poem, teaching, etc., possess generative devices that can introduce individual differences between one performance of the pragmeme and another. Within this framework, pragmemes provide space for individual freedom of action, enabling individuals to shape and reshape social rules to suit their needs. This characteristic is what distinctly positions pragmemes as a domain within linguistics that is intricately tied to the endeavor of safeguarding human rights and engaging in the negotiation of social and sexual identity through linguistic actions.

## 3 The interplay of pragmemes and culture

Speakers use their language creatively, up to a certain point. We are clearly able to understand novel utterances (those we have never encountered before) by using (being competent in) the lexicon and grammatical rules, which can be considered the semantic glue that creates utterances by putting syntactic constituents (NPs, VPs, APs, and ADVPs) together and allow us to distinguish between different logical forms by showing how different they are semantically and syntactically. Even if the understanding of language is, in general, based on conventions, language use plays a role in the sedimentation of meaning. By language use we mean the influence of context in utterance interpretation. The meaning of a word can become richer or poorer in context. A toy gun is less than a gun, for example. A horse in a game of chess is not a real horse but a piece subject to the rules of chess. However, if you say, “The surgeon has arrived,” you probably mean, “The male surgeon has arrived.” A narrowing of the NP denotation occurs due to inferences to stereotypes.

Pragmemes are speech acts in context. They carry out intentionality and compel hearers to interpret intentions correctly (if there should be a match between the speaker’s and the hearer’s intended meanings). Like language games, they follow certain societal rules, are bound to certain activities and constitute social activities. Take the case of law making. Laws constitute the fabric of society; without the laws, society, as we know it, could not exist and would soon disintegrate.

Pragmemes are chunks of social activity separated from other chunks by formal markers, which make a certain activity recognizable as such. These could also be called discourse markers and instantiate what Goffman calls “frames.” Walls, doors, and windows, but also certain linguistic indicators (The bill, please!) are ideal for separating a section of interaction from another. Macrostructures are like frames, but they are defined in a logical way through the notion of entailment. Each section of interaction entails a larger section (ordering food, e.g., entails having dinner in a restaurant). We could use and integrate all these ideas to understand what pragmemes are. But without the notion of culture we could never make sufficient progress. Every social activity is defined within a culture and, without its cultural underpinnings, we would be lost in the attempt to understand what is going on in the interaction. Culture shapes what we see, what we hear, and what we understand. As Kecskes (2019) noted, certain expressions, to be properly understood, need a notion of context and need to be embedded in culture. Given the enormous difficulties involved in the interpretation process, it is not surprising that speakers from different areas of the world will resort to literal meanings to resolve the communication process.

## 4 Kecskes on pragmemes and conventionality

Extensive research on pragmemes has provided compelling evidence that the interpretation and production of speech acts are deeply intertwined with the socio-cultural context in which they occur. A body of investigations on pragmemes by Kecskes (2016) has challenged the notion of *linguistic creativity* of Chomsky (1964). It refers to the ability to generate infinite sentences using a limited repertoire of elements and algorithmic procedures to combine them. In Chomsky’s view, the mechanical memory of utterances in typical socio-cultural scenarios (i.e., pragmemes or frames) holds little significance. What truly matters is the combinatorial power of the syntactic engine, which enables the generation of grammatical structures irrespective of their specific use.

However, when we analyze actual language use, a notable contrast emerges. Data suggest that we tend to be slightly original and creative due to the substantial utilization of prefabricated language units [Altenberg (1998) even claims that nearly 80% of our linguistic production can be considered stereotyped]. The importance of speech routines has been noted in previous research by authors such as Hymes (1962), Bolinger (1976), and Fillmore (1976). They have pointed out that a considerable portion of verbal behavior is made up of the use of formulaic language. By formulaic language, they mean multi-word expressions holistically stored and retrieved in memory rather than generated component by component with each use. Collocations, fixed semantic units, frozen metaphors, phrasal verbs, speech formulas, and idiomatic expressions can all be examples of formulaic language. This considerable stock of prefabricated units in mind is ready to be called upon when the need arises and is mainly the result of normalization, standardization, the emergence of shared expectations, and the development of common ground in a community.

Psycholinguistic evidence strongly supports the critical role of formulaic expressions and fixed formulas in the economy of speech production. These formulaic utterances ease the linguistic processing burden, not only because they are ready-made and require no effort from the speaker or hearer to assemble them, but also because their meanings are readily accessible during real-time production and comprehension [this aspect has been validated by the idiomatic principle, as endorsed by the Sinclair (1991)]. As a default processing strategy, formulaic expressions become the most salient option in speech production. Consequently, speakers predominantly rely on them when they communicate. Only when using specific formulas is not feasible, may individuals resort to the principle of open choice (such as when speakers of a *Lingua Franca* lack shared common ground). Individuals who share a particular language and belong to a linguistic community exhibit preferred ways of expressing meaning (Wray, 2002; Kecskes, 2007). As a result, we often hear variations in how people from different linguistic and socio-cultural backgrounds transmit opinions, ideas, beliefs, or suggestions. For instance, an Italian may use the formulaic expression “ho fatto la doccia” (lit. I *did* a shower), a French speaker may say “j’ai pris une douche” (lit. I *took* a shower), while an English speaker may opt for “I *had* a shower.” Each formulaic expression (differing in verb selection) conveys the same sense of “washing oneself in a shower.”

However, it is essential to acknowledge that frequency alone does not determine the identification of formulaic expressions. We must discern between two distinct categories: (i) sequences of words that commonly occur together in various contexts (e.g., routine formulas like “no problem” or “you know”); and (ii) groups of prefabricated expressions that hold psychological relevance for speakers in a community only because they have a high degree of association with a particular situation. Kecskes (2010) refers to the latter as Situational-Bound Utterances (SBUs). SBUs derive their meaning from usage conventions rather than linguistic conventions. They are prefabricated pragmatic units that emerge in standardized communicative situations, i.e., pragmemes. What sets SBUs apart from conversational routines or other common sequences of words is their situational load. Conversational routines are like versatile tools in a speaker’s toolbox, capable of serving various functions across different situations, such as expressing agreement, disagreement, seeking clarification, or changing topics. These routines are crucial in maintaining smooth and coherent conversations, allowing for predictable and efficient information exchanges. For example, phrases like “I see,” “You’re right,” or “Let us move on” can be used in various contexts to serve their respective functions. On the other hand, SBUs are more like “tailored garments,” meticulously crafted to suit specific situations. These expressions draw their significance not from their general speech function but from the particular context in which they are employed. For instance, expressions such as “License and booklet” uttered at a roadblock by a policeman, “How do you do?” during introductions, or “Welcome aboard” as a greeting to a new employee only holds meaning within well-defined individual circumstances. Clearly, according to us, these utterances are pragmemes, in that their meanings are conventionally determined by knowledge of the situation in which the interaction takes place. Kecskes introduces a fascinating concept of pragmeme, characterized by a unique and singular interpretation of the speech act. This exclusivity in interpretation arises due to the high conventionality of the discourse situation and the norms governing its usage.

The prevalent use of prefabricated formulas, whether specific to particular situations or employed repeatedly in more than one circumstance, should not be misconstrued as evidence that linguistic creativity does not exist. On the contrary, Kecskes asserts that linguistic creativity transcends the mere combination of words and units of meaning within syntactic boundaries. It extends beyond the sentence level and becomes a discursive phenomenon (Kecskes, 2013). In this broader sense, linguistic creativity manifests as the ability to ingeniously integrate prefabricated units with novel elements, generating *ad hoc* expressions that effectively convey communicative intentions and objectives. This creative interplay predominantly occurs when the speaker seeks to manipulate their message, exercising control over what they wish the audience to believe or understand. Kecskes (2016, 12) considers the following case:

Roy: – Is there something wrong, Susie?

Susie: – I am fine, Roy.

Roy: – I would have believed you if you had not said “Roy.”

In the given example (1), Roy perceives that something might bother Susie and decides to inquire about it. Susie responds with the formulaic expression, “I am fine.” However, despite using that formulaic expression, she intends to convey to Roy that everything is not actually fine. She accomplishes this by strategically including Roy’s name in her response. Susie demonstrates her *deliberate creativity* by incorporating a new element into formulaic expression to satisfy her communicative needs.

The approaches proposed by Kecskes and Mey diverge significantly insofar as the first is rooted in a socio-cognitive dialectical perspective on communication and pragmatics. This perspective effectively bridges the gap between social and individual aspects of communication, recognizing it as a dynamic process where individuals are influenced and actively contribute to shaping their social conditions. Central to this viewpoint is the understanding that individuals produce and comprehend language by drawing upon their most accessible and relevant knowledge, influenced by their prior experiences. Individuals are not merely passive recipients of social norms but actively engage with and shape them through communication.

Now, after this digression on SBUs, we have to ask if pragmemes can be of two types:

- Situational-Bound Utterances where the force of convention shapes the meaning of a certain expression.
- Utterances that are enriched in interpretation due to the influence of the context of use, but whose meaning is determined on the spot, not “*a piori*” as happens with Kecskes’ SBUs. In such cases, inferential pragmatics plays a crucial role and the inferences are more easily cancellable than those of situation-bound utterances.

Cancellability of an inference is important because, according to Grice, it seems to point to the fact that the inference is an implicature, that is to say a logical form enriched due to the influence of the context and taking into account (or reconstructing) the intentions of the speaker.

SBUs, of course, preserve intentionality, but they are expressed through the notion of convention, following the cultural prescriptions related to the society the speakers belong to.

So, the question now arises: is culture completely ignored by the interpretation process of pragmemes that are not SBUs? Our answer is: No. Cultural information goes into the process of understanding the utterance. When you ask: what time is it? And the hearer replies: The milkman has arrived. He probably means that it is about 8 o clock, the time when the milkman usually arrives to deliver the bottles of milk in front of your gate. But this works well for English culture, where there are milkmen, and bottles of milk opposite people’s doors, but not for Italian culture, where, unfortunately, there is no equivalent of the milkman (except for translations of texts of the past) and no bottles of milk in front of one’s door.

## 5 The pragmeme “*vieni?*” in the Italian school context

One of the most interesting examples of pragmemes we have had a chance to study was a case of classroom interaction. This type of interaction too is under pressure to evolve. In old times, the testing process (in Italian schools) occurred in a space of almost physical contact between the student to be tested and the teacher. Nowadays, the student can remain at his desk and answer from there the teacher’s questions. So, if the student is at his desk, he can choose to answer the teacher’s questions from there. The question “Vieni?” (Will you come?) has, therefore, somewhat changed in its meaning from a literal question as to whether the student is ready to be examined and is also willing to go to the teacher’s desk so that the proceeding could be carried out to a non-literal question. It amounts to a non-literal question as to whether the student is ready to be examined from his own optimal position or from the teacher’s position, where his close desk mates could be of no help in suggesting answers. The utterance “Vieni?” has lost its implications of motion toward the teachers’ desk, despite being still conventionally associated with the function of examining/being examined. This is clearly, as Kecskes would say, a Situational-Bound Utterance, as it works in the context of the class, at the strategic point in which the roll-call has been made, and possibly after the teacher has lectured on some topic. The position in the interaction and the discourse maker “Vieni?” help establish the conventional meaning/function of the utterance.

In what ways is this pragmeme related to culture? I remind readers that exams in Italy, in addition to having a modest written component, both at school and at the University level, unlike in many other countries, normally take an oral form, in line with an old oral tradition according to which the student had to exercise his memory and his rhetorical competence. Due to his mnemonic work, the Italian student can be brilliant in exposing in oral form the contents of his knowledge and also brilliant in answering complicated questions by the teacher, whose purpose is to test his critical competence, his synthetic qualities and, also, his abilities in problem solving.

## 6 Barack Obama’s rhetoric and pragmemes

In his article on Barack Obama’s South Carolina victory speech, Capone (2010e) examines some of the President’s most successful strategies in obtaining electoral consensus. The speech is often accompanied by utterances of “Yes, we can” (in response to the main speaker), which, on the one hand, vocalize enthusiasm for the President and, on the other, sums up the President’s philosophy of life, which, in turn, expresses American’s deepest beliefs. This utterance is closely related to culture, given that American history has often been guided by ideals such as change for the best, optimism, and the idea that anything (or almost anything) can be achieved by hard work and strong will. The utterance “Yes we can,” incorporates all civil conquests, such as the parity of the sexes, the fight for religious freedom, the fight for sexual liberation, the fight for equal access to rights, the parity in enfranchisement (which, however, was put in question after the election of Donald Trump), etc. These are the heritage of political conquests, for which Americans have paid dearly. They are certainly an important part of American history, civilization, and culture. For this reason, one could say that “Yes we can” counts as a pragmeme, given that, in isolation (if the literal import is considered), the utterance says very little, but it must be enriched from a cultural point of view to do the work it does in calling back to mind important chapters of American history and culture. This is why Wong (2010) talks about the triple articulation, given that, without reference to culture, the message of the pragmeme would be lost.

Capone sheds light on another interesting aspect of Obama’s speech, drawing attention to its polyphonic nature [for a more extensive discussion on “polyphony” see Bakhtin (1981, 1986) and Capone (2016)]. Obama frequently employs the technique of personification. Rather than merely presenting an idea as if it originated from himself, he gets another person (fictitious or, plausibly, real) to voice it. In the context of the electoral speech delivered in South Carolina, where ceremonial constraints precluded the presence of additional individuals on stage, Obama personifies ideas by recounting what people conveyed to him. Through this strategic approach, not only does Obama engage/address the audience as active participants in the speech event, but also reverses the direction of influence from the people in control to the people controlled (see Van Dijk, 2003). A more technical way to explain Obama’s strategy can be articulated by drawing upon categories of *animator*, *author*, and *principal* of Goffman (1981). The animator is the one who vocalizes a text without necessarily being its composer or the owner. The author is the individual responsible for composing the message to be conveyed, while the principal takes ownership of the position or opinion being asserted. While, in many instances, these three roles coexist within a single individual who is committed to advancing an argument, Goffman (1981, 128) observes that on specific occasions (e.g., theatrical work, conferences, poetry recitations, etc.) the speaker can undergo *a change in footing*. During these moments, the speaker may transition from the roles of principal and author to that of animator (although other role changes are also conceivable). Goffman highlights how these shifts in footing are precisely demarcated by both microscopic and macroscopic signals within a speech. These signals encompass body movements, gestures, tone of voice, facial expressions, or shifts in stylistic register. This observation is intricately tied to the discourse on polyphony. Whenever a speaker feels the need to change his position in the conversation (thereby incorporating the speech of others), he employs a combination of linguistic and extralinguistic strategies to effectively signal this change to his audience. In the specific instance under consideration, Obama skillfully uses pauses, variations in tone of voice, and evaluative devices. The latter are employed with the specific intention of articulating the judgments of others regarding one’s actions, facilitating a genuine identification with the various members of the public.

What is the relationship between footing and the pragmeme? We have proposed that the pragmeme is a speech act in context. While speech act theory takes for granted that the speech act has to be explained in terms of the intentions of the speaker, it does not say that the speaker is a laminated context for Goffman and that we need to specify whose speech act that utterance is. Surely there is a speech act and an utterance when someone speaks, but the purpose of the speech act need not be attributed to the speaker, as the speaker may be an intermediary and may speak for a different person. Thus, we need to know some clues that specify how to interpret the utterance and how to attribute intentions (by choosing the speaker or someone he speaks for).

We could say much more about this, but this would be like opening a window on a different paper. We reserve the right to address this issue, which is of importance, in a different place.

## 7 Other examples of pragmemes

We have always been fascinated by the substantial difference between the English “recommend” and the Italian “raccomandare.” The former would be suitable for a context in which the speech act “recommend” is done by someone who has the authority, the right or obligation to write a report on behalf of a candidate who needs such a letter, e.g., for a job application, competition, etc. Although the candidate has the right to ask, the referee could very well deny such a reference, but normally his obligation is to write it and, if he decides to do so, he has the duty to be truthful, up to the point, relevant, and not to provide misleading information. In Italian, the speech act “raccomandare,” usually refers to an act done for friendship, opportunism, or even the desire to help a candidate, regardless of the real value of such a candidate (so, a different verb must be chosen if these conditions are not met!). This may very well be due to the political events that have become part of the official culture and common ground. The differences between English culture and the Italian are not negligible and they really have an enormous impact on languages.

Another culturally loaded word is the English “patronize,” as in “You are patronizing me.” We take it up as an example part of the experience of one of us; when the person in question studied in Edinburgh, in the Hume Tower, one of his friends often said “You are patronizing me” and these words were rather obscure to the hearer, except for the fact that he realized he had said (and done) something which was taken the wrong way. With Italian people of your age, you can take for granted that you can give them advice, share with them your view of the world, reservations about their conduct, etc. Even if they do not take the advice, at least they will not blame you for that, and they will not object that you do not have the right to interfere with their lives. The English would resent most your attitude of superiority, that is to say, your view that you know more than them. Being in a knower state creates problems, even if you know what should be done in a certain situation. What does culture have to do with this? Presumably, Italians are more friendly and respect more one side of the maxim of tact (Be altruistic), whereas the English would solely pay attention to the negative face, that is they do not want to give the impression that their pieces of advice are embarrassing.

## 8 Shouting at the market-place

In Capone’s papers for “Lingua” (Capone, 2018) and for “Semiotica” (Capone, 2023), the author clarifies that, often, the poetic function of language combines with other functions, e.g., selling things, asking people to do things, etc. Whereas in the past, it was customary to see vendors shout at the marketplace and use poetic language to attract especially women so that they could observe and buy their merchandise; nowadays this has become a rare phenomenon, due to the financial crisis, and the fact that many foreigners are employed at the market place, so even if they wanted to implement the traditions, they could not, because they have not received sufficient training (We are not talking about the training of the formal type, but being immersed in the social practice, playing that language game).

From a formal point of view, in these types of discourse, you could find all the rhetorical devices of poetry, including rhythm, rhymes, alliteration, metaphor, and inversion (Signora, Signorina, Signorina, and Signora). And most surprisingly, you could find vendors from other stalls replying to your speech and hear some unusual, extravagant rhymes. This marks this type of speech as a polyphonic language game. This language game has some constants:

- the vendor represents himself as someone in need who must make incredible discounts to get rid of his merchandise;
- the vendor represents himself as being mad, as he sells his goods at a very low price;
- being humorous by inventing situations (look under the merchandise and you will find your husband with his lover) that trigger a search;
- comparing one’s merchandise with those of other vendors;
- flattering the clients;
- using hyperbolic language; and
- giving reasons for buying the merchandise.

These do not look like unskilled poets, but like accomplished poets who know well that, at the end of the day, their creativity will be rewarded. This activity is creative because it can be rejuvenated in all sorts of ways, say by adding tropes, by adding metaphors, and by using polyphony, that is to say by incorporating other people’s discourses into our own.

## 9 Trump’s order (or suggestion) to Comey

The concept of pragmeme holds significant importance in linguistic research, encompassing various crucial aspects. Unlike traditional approaches that prioritize literal meaning before pragmatic enrichments, the theory of pragmemes encompasses a unified perspective on communication. As Mey (2001) points out, utterance interpretation is a holistic process, and in many cases, we may already anticipate the meaning of an utterance even before hearing it. The overall situation and our sociocultural schemas can strongly influence the interpretation of what is being said. According to Mey (2001) and Capone (2005), a pragmeme is a speech act embedded in a context. Context, particularly the cultural one, assumes a paramount role in shaping the meaning and function of an utterance. The influence exercised by context is, in prevalence, top-down.

Pragmemes are interpretations of speech acts that rely on various contextual cues. The merge of these cues will result in a particular interpretation, making it prevalent and resistant to change. Within this context, it is beneficial to distinguish between two types of pragmemes. The first type consists of pragmemes with only one possible interpretation due to the high level of conventionality in the discourse situation and the linguistic usage. Akin to the “SBUs” (Kecskes, 2013) discussed earlier, these pragmemes follow well-established patterns, leaving little room for ambiguity. In contrast, the second type comprises pragmemes whose interpretations are not rigidly bound by the actual situation or linguistic conventions. Instead, their understanding relies on ample contextual cues and appropriate reasoning. These factors favor selecting the most plausible and certain interpretation while dismissing less likely alternatives.

In a paper on pragmemes theory, Capone and Bucca (2019) provide a practical application of the second type of pragmeme, focusing on an utterance by Trump addressed to FBI Director Comey: “I hope you will let Flynn go.” When examined in a hypothetical context, the utterance may be interpreted merely as an expressive or declarative speech act. However, considering the actual situational and contextual factors surrounding the utterance, particularly the roles of the agents involved, the interpretation is narrowed down to that of an order. To fully understand the intended meaning, it is essential to consider the institutional positions held by both Trump and Comey within the United States government. At the time of the statement, Trump served as the head of state and the leader of the American government, while Comey held the prominent position of the US FBI director. The fact that their interaction occurred in a public setting, implies that they acted in their official capacities. In democratic states like the United States, the principle of separation of powers is fundamental, preventing the executive branch (represented by the President) from interfering with the judiciary system. If such interference were to happen, it would lead to a constitutional crisis. Based on these premises, it becomes evident that individuals holding similar offices are not entirely free to choose what they intend to communicate. In the case of Trump’s statement to Comey, it is crucial to recognize that he was not speaking as a private citizen engaging in an informal conversation. Instead, as the President (in the attributive sense of the term), his words carried significant weight and potential consequences. Being aware of the implications of his linguistic actions, Trump should have exercised greater caution in his communication. “I hope you will let x go” takes on a distinct meaning (say an order) when spoken by an institutional office addressing another institutional office with independent and autonomous power. This awareness should have compelled the former to plan his statement meticulously to avoid negative interpretations. One effective approach to avoid any appearance of making an indirect request would have been to refrain from expressing hypothetical hope. Often expressing hope implies some level of implicit request, while adhering to a principle of caution would have been wise. Trump did not adopt such measures.

Furthermore, it is essential to consider the scope of authority and capabilities of both offices in question. While it is evident that the President of the United States cannot interfere with a judicial investigation, it is equally valid that the Director of the FBI possesses the power to terminate an ongoing investigation into an individual. This additional element holds significant importance because the context in which hope is expressed to individuals capable of fulfilling it differs significantly from expressing it to those who cannot. When we express hopes to individuals without the means to fulfill them, such expressions often function as genuine, expressive speech acts (e.g., I express hope of getting an excellent grade in math to my mother). However, in a situation like the Comey/Trump encounter, where a powerful individual addresses another with the authority to make the hoped-for event a reality, the expression of hope can readily be perceived as a request. The recipient may interpret the statement as an appeal to take action in bringing about the hoped-for outcome. Given the specific context of their interaction, neither the speaker nor the hearer can ignore the potential for the speech act to be interpreted as a request. Both parties should be fully aware of the implications of their words and actions, recognizing that the statement holds the weight of a request due to the power dynamics at play.

Further evidence supporting the interpretation of Trump’s utterance as an order can be derived from the potential consequences of other interpretative hypotheses. If Trump had merely expressed a genuine hope without any intention of influencing Comey’s conduct, his speech would have been inconsequential for both himself and Comey. It would lack any significant purpose that warrants the mobilization of linguistic resources and the inferential processes involved. However, the situation indicates otherwise. It is reasonable to assume that someone who is the subject of a judicial inquiry or may face negative consequences (especially regarding their public image) would strongly prefer to avoid such an inquiry altogether or have it halted.

The examined factors are intrinsically connected to pragmemes, as sociocultural constraints heavily influence the production and comprehension of speech acts. To fully grasp the meaning behind Trump’s statement, one must delve into the cultural and environmental context in which it was made. Had the same utterance been voiced in Italy or Turkey, different interpretations could have emerged, given the distinct cultural contexts that may not align with *that* specific interpretation. Similarly, an alternative interpretation could have been favored if the statement had been uttered in a different, non-political setting. The crux of the matter is that every speech act, whether ceremonial, informal, public, or private, undergoes shaping by the available frameworks, interpretive paths, norms, and environmental constraints. These elements act as filters, segmenting and directing the interpretative process. They work to exclude alternative pathways that might be theoretically possible but remain out of reach due to the specific combination of the discourse’s agents, historical moment, and context.

Trump’s case also raises a significant issue concerning speech act theory, challenging the notion that an explicit performative is always required to convey order (as Austin believed). Strawson, in contrast, clarified that speech acts do not rely solely on explicit performatives; instead, they depend on implicit linguistic resources, context, and pragmatic reasoning, including Grice’s conversational maxims. For instance, in a workplace scenario, my boss can simply say, “You can go,” and I would understand that he means, “I want you to go.” The use of mitigation and implicit resources can cancel a literal interpretation of a speech act and significantly lean toward a non-literal interpretation. Strawson’s perspective suggests that performatives are infrequently used, as the context often makes it evident how a speech act should be interpreted.

According to Strawson’s perspective, considering the alternative hypothesis that Trump merely made a straightforward assertion is difficult to defend. When the President states hope that P, he may express not just his desire for P to occur but also his belief that P is preferable to non-P. The crucial point here is that by publicly affirming his dialogical commitment to a public official like Comey, Trump could be seen as attempting to influence Comey’s behavior and implicitly communicating his belief that Comey’s actions will align with Trump’s hopes. Language is not simply a tool for conveying information but also a means of exerting influence over the attitudes and conduct of others. When we speak, our linguistic actions inherently aim to shape our listeners’ perceptions or actions in specific ways.

These situational elements converge to convey a specific and undeniable meaning, leaving little room for denial. While there can be much debate about the exact proposition that the hearer retrieves from a statement like “I hope you will let Flynn go” (and any explicatures that are part of it), Trump’s intention to influence Comey’s behavior is undeniable. Such an intention cannot be canceled without giving rise to discursive anomalies or meaningless speech. It is highly implausible to assume that Trump merely expresses hope while remaining neutral regarding whether Comey will fulfill that hope. Such an interpretation would be futile and wasteful, expending energy with no cognitive reward. We can safely assume that people do not engage in wasteful communication and express their propositional attitudes without intending to affect the interlocutor’s attitudes. The discussion highlights that in certain pragmemes, the literal meanings of expressions or linguistic conventions may take a less central position. Instead, what prevails is the presence of various clues and presuppositions, encompassing cultural, social, or environmental aspects that unequivocally guide us toward a particular interpretative paths, distinguishable from other potential interpretations.

## 10 Names as pragmemes

Capone (forthcoming) challenges the theory on proper names, by saying that the function of proper names is not solely to refer to a thing or an individual to which the speaking subject attributes a predicate in the course of asserting a proposition. Surely this is one of the uses of proper names, but very often proper names can be uttered in isolation and function as sentential fragments. Uttered in isolation, they can be used to call someone or an animal, to inhibit an action which is deemed to be inappropriate or pernicious or even to scold a child, an adult, or a dog. This paper expatiates on the distinction between addressing someone and calling someone and much of its interest lies in substantiating this difference. Here it is not possible to go into details. However, it is clear that proper names require contextualization for their understanding, whether they are used to refer to individuals or things or whether they are used to address someone, to call someone, to warn someone, or to scold someone.

## 11 The triple articulation of language

In recent years, the field of linguistics has undergone a significant transformation. Previously, there was a predominant focus, if not an exclusive one, on the formal aspects of language. This focus is derived mainly from the acceptance of the Chomskyan concept of “linguistic competence,” (Chomsky, 1965) which sees language knowledge as an innate, isolated ability divorced from real-world usage and cultural nuances. Chomsky’s concept of linguistic competence illustrates language proficiency as a purely structural phenomenon devoid of the influence of psychological and sociological factors. However, this perspective fails to capture the complexities of actual communication, as it ignores the situational appropriateness of utterances.

In response to these limitations, Hymes (1966) introduces the concept of “communicative competence.” Communicative competence recognizes that language cannot be analyzed as a static, uniform entity but should be viewed as a dynamic interplay of form, meaning, cultural norms, values, background knowledge, and contextual appropriateness. Unlike the rigid boundaries of linguistic competence, communicative competence acknowledges the ever-changing nature of language, shaped by many sociolinguistic factors. Hymes argues that to master a language, one must not only understand its grammatical structures but also develop the ability to apply this knowledge effectively in real-life situations. In his view, communicative competence encompasses evaluating one’s speech and choosing the most appropriate linguistic forms from myriad options. This selection process is finely attuned to the needs and expectations of the hearers in a given situation (Hymes, 1972).

The last point is essential in our discussion on pragmemes: while comparable linguistic structures exist across various languages, their uses and restrictions differ significantly within different cultural contexts. These variations are contingent upon the values upheld within specific communities. Consider the imperative construction “Open the window.” Structurally, it can be analyzed as a simple verb phrase, as it is centered around one verb. Semantically, it conveys the meaning “I want you to do something,” indicating the speaker’s desire for the addressee to take action (Wierzbicka, 1996). Similar structural and semantic interpretations can also be identified in other languages. However, *culturally*, these structures undergo several constraints when placed in specific situations. An example is observed by Wong (2010) in Anglo English. In Anglo-English culture, there is a reluctance to instruct others directly: avoiding the imperative in ordinary situations is a linguistic manifestation of the cultural value against imposing commands on people. This reflects a deep respect for individual autonomy (Brown and Stephen, 1987). Consequently, in Anglo-Saxon cultures, orders are often framed as questions or expressed indirectly, as in phrases like “Can you pass me the salt?” or “I hope you finish the work by Friday.” In contrast, in Middle Eastern, Slavic, Dravidian, and Southeast Asian cultures, using questions or indirect expressions to prompt someone to complete a task might be perceived as excessively polite or even odd (Wierzbicka, 1991, 2006; Gladkova, 2007; Wong, 2007). In these cultural contexts, the imperative form is the norm when instructing someone to do something. These variations underline that the pragmeme of ordering someone to perform a task also inevitably requires knowledge of cultural and societal norms, which in turn influence the correct use of linguistic forms.

Wong shares Hymes’ idea that the study of language must go beyond grammar and incorporate understanding of the cultural expectations woven into specific situations. However, he believes that a prevalent static viewpoint persists concerning how extralinguistic factors impact speech production and comprehension. This rigidity is particularly evident in perpetuating universal and culture-independent interpretative principles, such as Grice’s maxims or Brown and Levinson’s concept of politeness (1987). Few studies challenge the predictive value of these notions in cultural contexts beyond English, the language from which they originated.

For example, Keenan (1976) argues that the Gricean maxim of Quantity is cross-culturally variable and that there are circumstances where being uninformative does not create any implicature but is the norm. Keenan examines some interaction patterns typical of the Malagasy society, where speakers regularly provide less information than the conversational partner requires, although they have access to the necessary information. When this happens, the implicature does not hold since the speaker’s expectations of the interlocutor’s behavior are met. The reason for this phenomenon can be found in the value of information within Malagasy society.

On his part, Wong (2010) also discusses compelling evidence challenging the universality of the maxim of Quantity. For him, in Anglo-English, adherence to this maxim often manifests through linguistic strategies like attenuation: speakers intentionally de-intensify what they say through expressions such as *a bit, a fair bit, a little bit, rather, and sort of*, etc. This linguistic tendency arises from a cultural inclination not to overstate, reflecting the practice of understatement in Anglo-Saxon speech (Wierzbicka, 2006). However, this norm does not apply universally to all human cultures. Singaporean speakers, for instance, exhibit a contrasting tendency—they prefer using emotional and exaggerated language to convey their feelings. Emotional expression through language, including exaggeration, is widely accepted and even encouraged in Singaporean speech. Historical and cultural contexts further emphasize this variety. In ancient Greece, for example, celebrated playwrights like Aeschylus, Aristophanes, and Antiphanes employed hyperbolic language in theatrical works. They used phrases like “*ten thousand horses*” or “*a myriad of men*” to describe quantities far more minor in reality, contrasting sharply with the current Anglo-Saxon norms of modesty. In the ancient Greek context, these hyperbolic expressions served a crucial purpose: emphasizing heroic deeds, fostering a sense of belonging to a powerful empire, and nurturing a collective identity among the people (for a more extensive discussion see Colace, 2023).

Undoubtedly, determining the universality of maxims is a challenging endeavor. There is no denying that these principles are intricately connected to facets inherent in human rationality. Grice’s initial conceptualization posits that these maxims stem from thoughtful reflections on the rational behavior of individuals engaged in dialogue; that is, each participant is supposed to know the norms for efficient and appropriate use of language without any cultural mediation. In Grice’s view, maxims are part of our cognitive setup (at least, each of us is capable of working them out). They have to do with the implicit knowledge of a set of principles and rules that govern non-linguistic behavior and, as such, represent the hallmark of human rationality. For example, if we interpret the maxim of Quality and Relation as an injunction to produce sincere and proportionately adequate acts, then we would expect that when we ask for a bottle of water at a bar, the bartender will not give us a bottle of wine. Similarly, in the wake of the maxim of Quantity, if we ask for three bottles of water, we will expect the bartender not to give us one or two of them. We also expect him to give us the bottles closed, with the cap pointing upwards, within a short time, and so on. One expects that any deviation from rational behavior will be interpreted as non-cooperative or insane—unless the bartender cannot fulfill our request; e.g., he does not have a sufficient number of bottles of water to fulfill our request, he is distracted, or there is a long line of people before us.

The existence of deviations from this ideal behavior does not invalidate the fundamental Gricean premise that communication is a rational endeavor. Nevertheless, it becomes clear that the concept of rationality requires careful “contextualization” when delving into the theory of pragmemes. It is no longer merely the ability to adhere to “universally” valid and good criteria; rational behavior boils down to what is most advantageous or productive within a specific community or context. In this perspective, being truthful, exhaustive, or relevant does not always yield advantages. Surprisingly, being uninformative or more informative can carry a certain utility and is, therefore, rationally justifiable. This has been demonstrated by cases documented by scholars like Keenan and Colace. However, we can discover rational justifications for deviating from the maxim of quality without needing to venture too far in time and space. Consider the phenomenon of “white lies,” where falsehoods are told not to deceive but rather to please or protect someone’s feelings.

Looking beyond the intricate matter of the rationality behind Grice’s maxims, the contributions of Hymes and Wong hold significant merit for presenting a more intricate understanding of communication dynamics. When exploring the cultural perspective of pragmemes, it becomes imperative to abandon the idea of making universal predictions about language. Specifically, we must get rid of the bias that assumes “what holds true for English speakers must apply to people in general” (Wierzbicka, 1991, 25). To achieve this, we need to move beyond mere form and meaning and delve into the cultural context within which language is employed and the pragmeme takes shape. Several studies have convincingly demonstrated that language is more than a medium for expressing meaning. It also, consciously or subconsciously, conveys culture—encompassing values, attitudes, and prejudices. Therefore, in the study of pragmemes, it is crucial to examine language within the cultural framework where it is employed. This comprehensive approach involves analyzing the dynamic interplay between form, meaning, and culture, acknowledging the subtle ways in which language reflects and influences cultural beliefs and practices.

## 12 Oishi on pragmemes and speech acts

Oishi’s (2016) work highlights that the situatedness of speech/pragmatic acts extends beyond the social, physical, and cognitive aspects to encompass the discursive situation in which the illocutionary act produces its effects. Mey (2001, 211) provides a characterization of pragmatic acts, which can be summarized as follows:

1. For sequences… to “count as” a pragmatic act, the circumstances (the “setting up”) must be right.
2. There need not be any speech act involved (of either bribing, making a request, or whatever else); it is the context that determines the nature of the pragmatic act.
3. Without “uptake,” there cannot be a pragmatic act; however, the uptake can be canceled by another, subsequent act.

As stated in (1) and (2), situatedness is crucial in defining pragmatic acts. They must be situated to be effective and actively contribute to creating the context in which they occur. In this passage, Mey deviates from Austin’s theory for two primary reasons: (i) pragmatic acts are not confined to specific speech acts, and (ii) they can encompass actions like “seeking compliments” or “soliciting an invitation,” which lack direct counterparts in speech acts and can also be expressed non-verbally. Despite these differences, Oishi posits that Mey’s concepts of situated speech acts and pragmatic acts share similarities with Austin’s original concept of speech acts.

According to Oishi, many scholars mistakenly perceive Mey’s theory as incompatible with Austin’s. This perception stems from misinterpreting some critical points in Austin’s theory. First, Austin does not rigidly define speech acts based on specific criteria; instead, he acknowledges that the number of acts depends on how finely we draw distinctions between them. For instance, is the illocutionary act of promising distinct from the act of swearing? Is the act of promising with authority different from a regular promise? Secondly, Austin’s locutionary, illocutionary, and perlocutionary acts are intended to elucidate various sources and effects of language use, not to classify real-life speech acts into fixed categories rigidly. These aspects of Austin’s speech act theory often go unrecognized, mainly because theory of Austin (1975) is frequently equated with Searle’s work. As Sbisà (2001:1795) points out, “Since Searle (1979, 46–49), the illocutionary act has generally been conceived as the act a speaker successfully performs when, uttering a sentence with a certain intention in certain circumstances, he or she gets the hearer to understand his or her intention.” In contrast, Austin does not allow the speaker’s intention to play an unlimited role in the successful performance of an illocutionary act.

The alignment between Mey’s conceptualization of situated speech acts and Austin’s notion of the felicity of speech acts lies in their shared emphasis on context. Austin lays the groundwork for the *conditions* that render acts situated, while Mey delves into the actual and prototypical circumstances in which such acts may be placed.

In exploring Austin’s felicity conditions, Oishi presents the following key points:

*The illocutionary act brings about its conventional effect when (i) the speaker, the hearer, and the circumstances of the speech situation are assumed to be the addresser, the addressee, and the context of the act, respectively [felicity conditions (A.1) and (A.2)], (ii) the speaker follows the procedure correctly (felicity condition (B.1)), (iii) the hearer ratifies the act (or the speaker makes a specific sequel) for the procedure to be completed [felicity condition (B.2)], (iv) the speaker has the thought or feeling, or intention of the addresser of the act (felicity condition(Γ.1)), and (v) the speaker or the hearer conducts her/himself subsequently as is specified for the addresser/addressee of the act (felicity condition Γ.2)* (Oishi, 2016, 338).

On his part, Mey argues that speech acts must be situated to be effective, emphasizing two aspects: (i) the alignment of the speaker, the hearer, and circumstances of the situation with the addresser, the addressee, and the context of the illocutionary act, strengthening the convention for producing the illocutionary effect; and (ii) the “affordability” that situational location allows. This enables a wide range of utterances with diverse lexical-semantic content to perform illocutionary acts and facilitates verbal allusion. Additionally, nonverbal acts can replace illocutionary acts, exemplified by the act of voting performed by raising one’s hand, and nonverbal acts executing actions not possible verbally, such as bribery (silences are also of interest).

Not only does Austin’s speech act theory provide insights into the linguistic aspects of communication but also enables profound social analyses of language use. When a speaker considers himself, the hearer, and the situational context as the addresser, addressee, and context of the speech act (pragmeme) he wishes to perform, the inquiry becomes both a linguistic and social endeavor. As Mey emphasizes, illocutionary act types (pragmemes) serve as situational prototypes, guiding individuals to adapt their speech acts (pragmatic acts) to real-life situations. Similarly, Austin’s notion of illocutionary acts can be analyzed as general situational prototypes, encompassing particular addressers, addressees, and contexts in which various illocutionary acts come into effect.

Oishi provides a clarifying example to illustrate this point. During a press conference in Paris on November 23, 2015, British Prime Minister David Cameron stated, “I firmly support the action that President Hollande has taken to strike ISIL in Syria.” Here, we can interpret this utterance in two ways: as the illocutionary act of introducing the topic of attacking ISIL or agreeing with President Hollande’s decision for France to strike ISIL in Syria. If the act of introduction is successful, David Cameron’s expression acquires the discursive status of introducing a new topic, leading to the interpretation of approval of the attack on ISIL. On the other hand, if the act of agreement is successfully executed, the expression acquires the discursive status of agreeing with President Hollande’s decision, resulting in the interpretation of support for France’s action against ISIL in Syria. The effectiveness of the illocutionary act hinges on the speaker’s acting as the addresser introducing a new topic, on the audience’s acting as the addressee accepting the introduction, and on the circumstances of the speech situation as the context for introducing the new topic. The critical takeaway from Oishi’s analysis is that the effectiveness of explicit expositive acts in discourse significantly influences how the present utterance is understood within the ongoing conversation. Depending on whether the act of introducing or agreeing is successfully carried out, the utterance *gains distinct discursive statuses*, leading to different interpretations of its meaning and intent. In this way, both the speaker and the audience co-construct the discourse.

To us, it is clear that, despite similarities, there is an obvious difference between the speech act and the pragmeme. When scholars (philosophers of language or linguists alike) discuss the speech act, they usually exemplify the speech acts by using simple sentences, which on the road toward contextualization, become full-fledged utterances, which express a certain function. The pragmeme is not necessarily a one-sentence utterance but may consist of several sentences strung together, in which each sentence takes on a different rhetorical role (e.g., main position, justification). It is true that scholars who defend the idea of the speech act may adapt to this holistic view of the text by saying that a speech act may consist of one or more utterances, even if for simplicity of discussion, they usually exemplify their notions through one sentence at most. Now, it may be true that the issue can be approached this way. But the fact that the context is often removed from the discussion of the speech act and that complex speech acts are omitted from the discussion points to a serious methodological flaw. This is not the way to do things, to discuss things, or even to test the theory. Austin came close to the notion of the pragmeme when he discussed examples like “I declare you man and wife.” Here he makes reference to a procedure that must be completed for the speech act to be successful. It follows that the utterance works inside that procedure and is conjoined with other utterances which makes the procedure correct.

A point of contact between the theory of pragmemes and that of speech acts is that in the cases of indirect speech acts, metaphoric utterances, or ironic utterances, the context has a top-down influence over semantic/pragmatic interpretation. One must know what the context is like even to start an analysis of the case. Mey believes that in the case of the pragmeme, context plays a top-down process, but it is clear to us that in such cases both bottom-up contextualization and top-down contextualization both play a role in utterance determination.

Oishi’s reflections are undoubtedly fascinating and highlight an important point long neglected in the analysis of speech acts: the speaker and the hearer are equal contributors to the creation of situated linguistic acts. Mey also emphasized the dynamic nature of the conversation. However, he placed excessive emphasis on the role of social contexts and situations without looking too much at individual dispositions. This “weakness” in the pragmeme theory was highlighted by Kecskes (2010), who recognized that individuals are not only bound by social conditions, but they shape these at the same time. Individual dispositions and previous experiences are decisive in making an utterance take on a certain discursive status. When individuals participate in a conversation with other individuals, the words and utterances they use are selected and formulated in relation to their first-person experiences. This means that a conversation is a unique encounter between individuals and social factors. To explain this concept, let us suppose that two children are making up a game. One says “caught.” The other stops at the place where he is. Another says “Liberated,” and the child who has been caught escapes, running. They are not just using rules, but they are inventing them. In some cases, students tend to erode rules or extend them. Rules are inherited by them from conventions. But the pressure of society is such that these rules cannot be kept as they are, thus they are changed somehow. Thus, it often happens that it is not only the teacher to criticize students, but the students can start criticizing teachers. It may also happen that not only teachers assign marks but that a teacher can ask the students to self-assign a mark and allow a discussion on this mark. Educational discourse is the place where changes occur more frequently, as students are not so happy to abide by the current rules.

Instead of speaker- or listener-oriented pragmatics, we need a pragmatic approach that offers a cognitive explanation. A speaker will always try to use those utterances that he thinks will best convey his/her intentions in a given situation, and vice versa, a hearer will always rely on those first-person experiences with the felt linguistic objects that he thinks best connect to the utterance of the speaker in a given situation. In this sense, the new frontier of cognitive studies can fill the gap in the cognitive structures that favor specific conceptual and communicative skills in complex and dynamic communicative contexts where situated speech acts occur.

## 13 Conclusion

We live in a world in which the notion of the pragmeme competes with other notions, such as that of the speech act. We have thought that, by extending the examples of Capone’s (2005) paper, we could have a more circumscribed and orderly theory of pagmemes.

It would be wise to follow Jock Wong’s instruction to proceed toward a cultural perspective that sees language and culture intertwined. This will give a mine of new examples, whose discussion may lead toward a revision of the theory.

## Author contributions

AC: Writing – original draft, Writing – review & editing. RG: Writing – original draft, Writing – review & editing.

## In memoriam

This paper is dedicated to the memory of Jacob L. Mey.
